# Multifunctional Ionogels Incorporated with Lanthanide (Eu^3+^, Tb^3+^) Complexes Covalently Modified Multi-Walled Carbon Nanotubes

**DOI:** 10.3390/polym10101099

**Published:** 2018-10-05

**Authors:** Qiuping Li

**Affiliations:** School of Materials and Chemical Engineering, Ningbo University of Technology, No. 201, Fenghua Road, Jiangbei District, Ningbo 315211, China; liqiuping@nbut.edu.cn; Tel.: +86-574-87081240

**Keywords:** lanthanide complex, ionic liquid, carbon nanotube, ionogel, composite materials, luminescence

## Abstract

Ionogels refer to an emerging composite material made from the confinement of ionic liquids within some specific cross-linked network matrices. They have potential applications in areas such as electrochemical and optical-electric materials. Incorporation of lanthanide (Eu^3+^, Tb^3+^) complexes covalently functionalized multi-walled carbon nanotubes (MWCNTs) in ionogels provide new ideas to design and synthesize novel luminescent hybrid materials that have excellent characteristics of luminescence and ionic conductivity. Here, the multifunctional ionogels were synthesized by confining an ionic liquid and the rare earth functionalized MWCNTs in the cross-linked polymethyl methacrylate (PMMA) networks, resulting in a novel optical/electric multifunctional hybrid material. The SEM images and digital photographs suggest that the lanthanide functionalized MWCNTs are evenly dispersed in the hybrid matrices, thus leading to a certain transparency bulky gel. The resulting ionogels exhibit certain viscosity and flexibility, and display an intense red/green emission under UV-light irradiation. The intrinsic conductibility of the embedded ionic liquids and carbon nanotubes in conjunction with the outstanding photoluminescent properties of lanthanide complexes makes the soft hybrid gels a material with great potential and valuable application in the field of optical-electric materials.

## 1. Introduction

Over the past decades, a number of researchers focused on the design and synthesis of photofunctional hybrid materials which were based on lanthanide compounds given that they have a wide range of applications in areas such as chemical sensing, bio-imaging, and organic light-emitting devices (OLEDs) [1,2,3,4]. The combination of lanthanide compounds with variable matrices (silicon, carbon, zeolite, polymer, etc.) provide an excellent opportunity to ensure the functional entity between the intrinsic characteristics of hybrid hosts (such as fabricability, mechanical strength, conductivity, thermal and chemical stability, and bio-compatibility) and the outstanding luminescent properties of lanthanide compounds (such as line-like emission, long luminescent lifetime, large Stokes shifts, and high luminescent quantum yield) [5,6]. Among these lanthanide functional hybrid materials, one is featured in chemical assembly in which the lanthanide complexes are linked onto some matrix moieties through strong interactions such as covalent, coordination, or electrovalent bond, having drawn much more attention than those physical doped hybrids [7]. This is due to the fact that the chemical assembly of lanthanide hybrids can overcome the disadvantages of concentration quenching, clustering of emitters, inhomogeneous property, or poor chemical stability. Thus, numerous lanthanide hybrid materials are synthesized based on this method, which has proved to be highly efficient for constructing homogeneous luminescent materials [8,9,10,11]. 

Gelatinous materials obtained from the gelation or confinement of ionic liquids are routinely called ionogels [12,13]. These kinds of soft materials can be easily prepared via the gelation effect of specific dicationic organic salts in ionic liquid solutions [14,15] or through the confinement of an ionic liquid within some cross-linked silica, polymer, or metal-organic framework matrices. [16,17] The wide electrochemical window property, high temperature adaptation capacity, along with other characteristics of ionogels, have generated great interest from material researchers from a variety of fields [18,19]. Moreover, researchers have proved that the gel system has great capacity for assembling multifunctional materials by incorporating some functional nanoparticles [20,21]. Hence, the combination of ionogels and functional nanoparticles may result in a synergistic property enhancement, and it has the potential of providing multi-functional hybrid materials with novel applications. It has been proved that ionic liquid-based hybrid materials have broad application futures in the fields of electrochemical sensing, supercapacitor, and luminescent materials [22,23]. Therefore, the combination of lanthanide complexes functionalized nanoparticles and ionogels provide new ideas to design and synthesize novel luminescent hybrid materials that have excellent characteristics of luminescence and ionic conductivity. Although many works have demonstrated that the incorporation of lanthanide complexes into the confined ionic liquids system could result in hybrid materials with intense photoluminescent properties [24,25,26,27,28], it is still pertinent to carry out further research into the synthesis and characterization of functionalized nanoparticles composite ionogels.

Carbon-based matrices, such as carbon dots and carbon nanotubes (CNTs), were usually selected as supported matrices for lanthanide complexes during the preparation of lanthanide-based photoluminescent hybrid materials [29,30,31,32]. For example, Dai et al. [33] argued that the carbon dots and Eu^3+^ complexes were homogeneously fabricated into monolithic ionogels with bright white photoluminescence. Zhao et al. [34] reported a novel luminescent Eu^3+^ complex functionalized single-walled carbon nanotube which showed the sensor properties of DNA sequence-dependent red luminescence enhancement. This research is inspired by these studies, and it aims to demonstrate the synthesis and characterization of two kinds of composite ionogels which were incorporated with the lanthanide complexes covalently modified MWCNTs as the luminescence center. The resulting materials due to their photophysical properties, were investigated in detail. 

## 2. Materials and Methods 

### 2.1. Materials

Aladdin reagents Co. LTD (Shanghai, China) provided all the required chemicals, including: 2,2′-bipyridyl-4,4′-dicarboxylic acid (96%, BPC), *N,N*′-dicyclohexylcarbodiimide (99%, DCC), 4-dimethylaminopyridine (99%, DMAP), azodiisobutyronitrile (98%, AIBN), methyl methacrylate (99.5%, MMA), 2-thenoyltifluoroacetone (98%, TTA), and trifluoroacetylacetone (98%, TFA). Amine modified-MWCNT (MWCNT-NH_2_, OD: 8–15 nm, 95 wt %, NH_2_ Content: 0.45 wt %) was purchased from Beijing Boyu Gaoke New Material Technology Co. Ltd (Beijing, China). 1-butyl-3-methylimidazolium bis[(trifluoromethyl)sulfonyl]imide (99%, [BMI][BTSI]) was provided by Shanghai Cheng Jie chemical Co. LTD (Shanghai, China). The EuCl_3_**·**6H_2_O and TbCl_3_**·**6H_2_O were prepared by dissolving the corresponding metal oxides in concentrated hydrochloric acid, afterwards, the respective lanthanide salts were collected by directly evaporating and crystallizing the solution. The other reagents were analytically pure and provided by the Sinopharm Chemical Reagent Co. (Shanghai, China).

### 2.2. Characterizations

Infrared (IR) spectra were obtained on NICOLET iS50 using KBr pellets for a solid sample. The thermogravimetric (TG) analysis and differential scanning calorimeter (DSC) were measured with Netzsch STA 449 C (Ningbo University of Technology, Ningbo, China) at a heating rate of 15 °C min^−1^ under a nitrogen atmosphere. The scanning electron microscopy (SEM) images were obtained using a Phenom Pro-SE instrument (Ningbo University of Technology, Ningbo, China). The luminescent excitation and emission spectra were recorded with the Hitachi F-4600 instrument (Ningbo University of Technology, Ningbo, China), while the luminescent lifetimes were obtained using the Edinburgh Instrument FLS920 (Tongji University, Shanghai, China). The rare earth elements analysis was measured on the Shimadzu atomic absorption spectrophotometer AA-6880 (Ningbo University of Technology, Ningbo, China). The electrochemical impedance spectroscopy (EIS) analysis was performed with the Gamry CHI660E instrument (Jiangsu University, Zhenjiang, China); the sample was sandwiched between two copper electrodes with an area of 1.5 cm^2^ and the thickness of the hybrid ionic liquid was calculated to be about 2 mm.

### 2.3. Preparation of Lanthanide Complexes Functionalized MWCNTs

The precursor complex Eu(TTA)_3_(H_2_O)_2_ was first prepared according to the conventional route [35], as follows: 3 mmol TTA was dissolved in 10 mL ethanol under stirring and was kept at 60 °C and then the pH value was adjusted to 8.0 with a 2 M sodium hydroxide solution. Next, 1 mmol EuCl_3_**·**6H_2_O solution in ethanol was added to the mixture dropwise. After that, the solution was kept at 60 °C and stirred continuously to ensure a complete coordination. After removing the solvent by reduced pressure distillation, the residual was re-dissolved with 10 mL dimethyl formamide (DMF) and then filtered to remove the insoluble sediment. Then, the DMF solution of Eu(TTA)_3_(H_2_O)_2_ (solution 1) was collected and reserved for the next step without any further processes.

Furthermore, 1 mmol BPC in the anhydrous solvent of DMF and dichloromethane (DCM) was activated with DCC (1.5 mmol) and DMAP (1 mmol) for 1 h in the ice bath. Next, 100 mg MWCNT-NH_2_ was added into the solution and treated with supersonic dispersion for 10 min. The resulting mixture was stirred for 24 h at 40 °C. Afterwards, it was filtered through a microporous nylon66 membrane and washed with hot DMF repeatedly. The bipyridine moiety modified MWCNTs, also denoted as MWCNT-BPC, were collected and dried overnight in a vacuum oven. Finally, 50 mg MWCNT-BPC was dispersed into solution 1 for ligand exchange. To ensure that the reaction was completed, the mixture was first treated with supersonic dispersion for half an hour and then stirred for 24 h at room temperature. The lanthanide complexes functionalized MWCNTs were collected via filtration, washed with sufficient ethanol, and dried in a vacuum oven to produce MWCNT-Eu. The synthesis of MWCNT-Tb was very similar to the method described above for MWCNT-Eu. 

### 2.4. Preparation of Lanthanide Complexes Functionalized MWCNTs Doped Luminescent Ionogels

The MWCNT-Eu/MWCNT-Tb (2 mg) was evenly dispersed into 500 mg [BMI][BTSI] in a glass vial with the ultrasonic instrument. After that, the other half amount of 500 mg MMA was added into the solution. Then, a portion of AIBN (25 mg) was dissolved into the mixed solution under ultrasonic treatments (10 min) at room temperature. The system was kept in the ultrasonic instrument, and the temperature of the ultrasound bath was increased to 75 °C to produce a transparent and viscous liquid, which was then put into a 40 °C air-dry oven for a week for further aging. The flexible, transparent and viscous monolithic ionogels were gently removed from the glass vial, which is denoted as MWCNT-Eu-Ionogel and MWCNT-Tb-Ionogel respectively. The content of rare earth ions was determined with the atomic absorption spectrophotometer AA-6880 (Ningbo University of Technology, Ningbo, China). The elements (Eu/Tb) content could convert into the form of its corresponding complexes (Eu(TTA)_3_BPC/Tb(TFA)_3_BPC) in the ionogels, which were respectively 0.0892 and 0.0726 mg/g.

## 3. Results and Discussion

**Figure 1:**
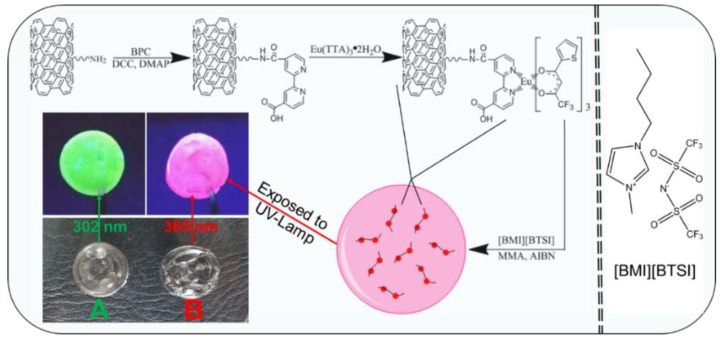
Synthetic procedure and predicted structure of the Eu^3+^ complex functionalized MWCNTs (multi-walled carbon nanotubes) doped ionogels. The digital photos of the resulting MWCNT-Tb-Ionogel (**A**) and MWCNT-Eu-Ionogel (**B**) under daylight (**bottom**) and UV-light (**top**).

In this work, lanthanide (Eu^3+^/Tb^3+^) complexes covalently functionalized multi-walled carbon nanotubes were first synthesized through a molecular bridge which was derived from the coupling reaction between the BPC and the amino-group carried on the surfaces of the MWCNTs. Afterwards, they were incorporated evenly into a [BMI][BTSI] ionic liquid system confined by the PMMA networks, thus resulting in two highly photoluminescent materials. Figure 1 shows the detailed synthetic pathways and how to obtain the ionogels, and their digital photos under daylight and UV lamp. The SEM images (Figure S1, Supplementary Materials) and digital photographs (Figure 1) suggest that the lanthanide functionalized MWCNTs are evenly dispersed in the hybrid matrices, thus leading to certain transparency bulky gels. The ionogels were flexible and viscous, and they showed intense red/green emissions (Figure 1) when they were exposed to the 302/365 nm UV light. 

**Figure 2:**
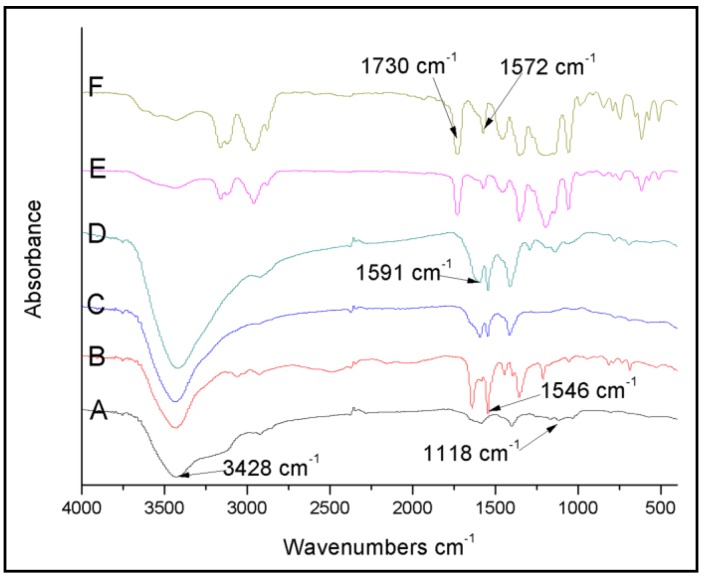
FTIR spectra of MWCNT-NH_2_ (**A**), MWCNT-BPC (**B**), MWCNT-Eu (**C**), MWCNT-Tb (**D**), MWCNT-Eu-Ionogel (**E**), and MWCNT-Tb-Ionogel (**F**).

The modification of the MWCNTs have been confirmed by the FTIR spectroscopy (Figure 2). The absorption band at 3428 and 1118cm^−1^ of MWCNT-NH_2_ (Figure 2A) was assigned to the N–H stretching vibration and the C–N stretching vibration, respectively. By comparing the spectrum to MWCNT–NH_2_, the 1546 cm^−1^ adsorption of MWCNT-BPC (Figure 2B) was assigned to the ring breathing vibration of the pyridine moiety, which could be seen as a sign of grafting success of BPC to the MWCNTs. However, the adsorption band around 1590 cm^−1^ of MWCNT-Eu (Figure 2C) and MWCNT-Tb (Figure 2D) is assigned to the C=O stretching vibrations of the chelate ring of the lanthanide complexes moiety. This indicates that the lanthanide was successfully assembled onto the MWCNTs. However, because the content of lanthanide complexes functionalized MWCNTs are almost insignificant in the final ionogels, it is impossible to detect their FTIR signals in the ionogels without enrichment. The adsorption band appearing around 1730 cm^−1^ of MWCNT-Eu-Ionogel (Figure 2E) and MWCNT-Tb-Ionogel (Figure 2F) is associated with the –COO– stretching vibration of the PMMA molecules, while the band for the ring stretching of the imidazolium moiety of ([BMI][BTSI]) is found at 1572 cm^−1^. These signals indicate that the PMMA and ionic liquids were incorporated into the resulting materials. 

**Figure 3:**
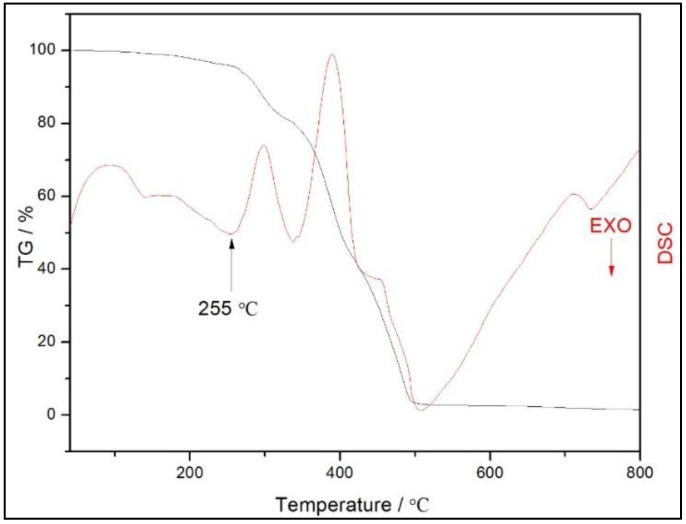
TG (thermogravimetric) and DSC (differential scanning calorimeter) analyses curves of the MWCNT-Eu-Ionogel.

To investigate the thermal stability of the composite ionogel, TG and DSC analyses curves of the MWCNT-Eu-Ionogel (Figure 3) were carried out under a nitrogen atmosphere at a heating rate of 15 °C min^−1^. It is noteworthy that the initial temperature for decomposing the sample is up to 255 °C, implying that the ionogels have good thermal stability. Below this temperature, the first weight loss of about 4.0% occurred at 40–255 °C, and can be attributed to the loss of some small molecules (physically absorbed water, residual MMA, and so forth). After that point, the major mass loss processes occurred until the ionogel nearly disappeared. Because the doped rare earth element is of a minimal amount, there was scarcely any remnants when the sample was heated to a high temperature.

**Figure 4:**
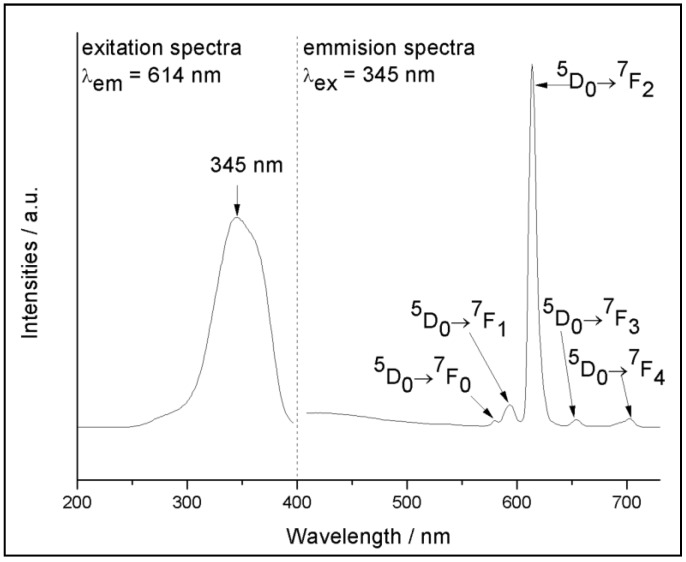
The excitation (**left**) and emission (**right**) spectra of the MWCNT-Eu-Ionogel.

Figure 4 (left) presents the excitation spectrum of the MWCNT-Eu-Ionogel, which was measured at room temperature by monitoring the characteristic luminescence intensity of the ^5^D_0_→^7^F_2_ transition of Eu^3+^ at 614 nm. As shown in the figure, the hybrid material exhibits an intense broad excitation band which centers at 345 nm. This mainly originated from the π→π* transitions of the ligand TTA, thus suggesting that an effective ligand-to-metal energy transfer took place in the ionogel. Figure 4 (right) also displays the emission spectra of MWCNT-Eu-Ionogel which was recorded under the excitation of a 345 nm wavelength. As a result, a series of typical transition bands were observed at 580, 594, 614, 654, and 703 nm, and were attributed to f–f transitions of ^5^D_0_→^7^F_J_ (J = 0, 1, 2, 3 and 4), respectively. The most intense emission (614 nm) originated from the ^5^D_0_→^7^F_2_ transition which belongs to the so-called hypersensitive transition, that is responsible for the red appearance of the ionogel under the 365 nm UV lights (Figure 1B). In addition, the ^5^D_0_→^7^F_1_ transition is a parity-allowed magnetic dipole transition which is relatively independent of the local environment of Eu^3+^ ions. Therefore, the comparison of the emission intensity between ^5^D_0_→^7^F_2_ transition and ^5^D_0_→^7^F_1_ transition can be seen as an indicator of the local environment of Eu^3+^ ions [36]. Here, the intensity ratio of the ^5^D_0_→^7^F_2_ to ^5^D_0_→^7^F_1_ for the europium-doped ionogel is 20.2, a value which indicates that the local surrounding around the Eu^3+^ ions in MWCNT-Eu-Ionogel is highly asymmetric. Furthermore, the luminescent decay curve of this sample was measured and the lifetime value (τ) was calculated as 527.3 µs (λ_ex_ = 345 nm, λ_em_ = 614 nm). The luminescent quantum efficiency of this ionogel was calculated as 56.06 % which is based on the ^5^D_0_ excited state of Eu^3+^ ions (detailed calculation processes can be seen in the supplementary materials). These results show that the hybrid system is an excellent host for the europium complexes.

**Figure 5:**
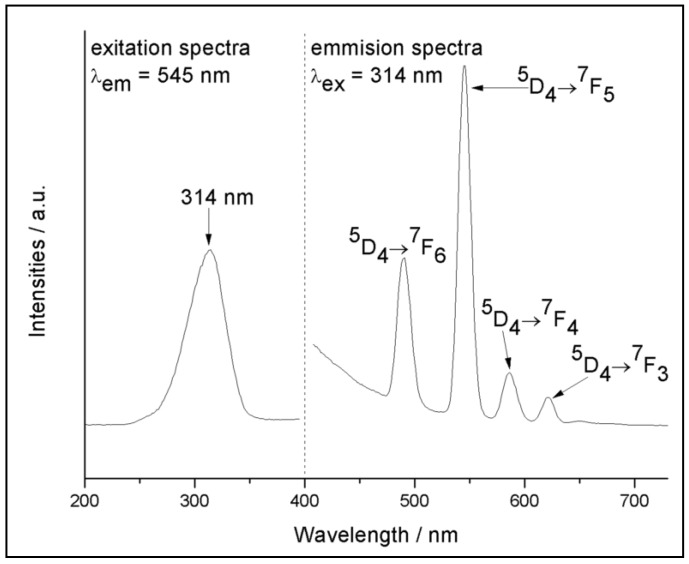
The excitation (**left**) and emission (**right**) spectra of the MWCNT-Tb-Ionogel.

Similarly, the luminescent excitation and emission spectra of the MWCNT-Tb-Ionogel were measured and presented in Figure 5. Here, the excitation spectrum (Figure 5 left) was recorded by monitoring the characteristic ^5^D_4_→^7^F_5_ transition of Tb^3+^ at 545 nm, which also possessed an intense broad band centered at 314 nm and was rooted from the Ligand TFA. Then, the corresponding emission spectra (Figure 5 right) was measured by using the 314 nm wavelength as the excitation source. There are four emission peaks which center at 487, 545, 582, and 621 nm, respectively, and they can be assigned to the corresponding ^5^D_4_→^7^F_j_ (J=6, 5, 4, 3) transition of Tb^3+^ ions. As shown in the figure, the dominant transition band is observed at 545 nm, and that is why the terbium-doped ionogel has a brilliant green look when it was exposed to 314 nm UV light (Figure 1A). The luminescent decay curve of MWCNT-Tb-Ionogel was also measured by using the 314 nm wavelength as its excitation source. The resulting luminescent lifetime value was calculated to be 507.1 µs. These findings are further proof that effective energy transfer has taken place during the process, thus suggesting that the hybrid system is an excellent host for the lanthanide complexes.

**Figure 6:**
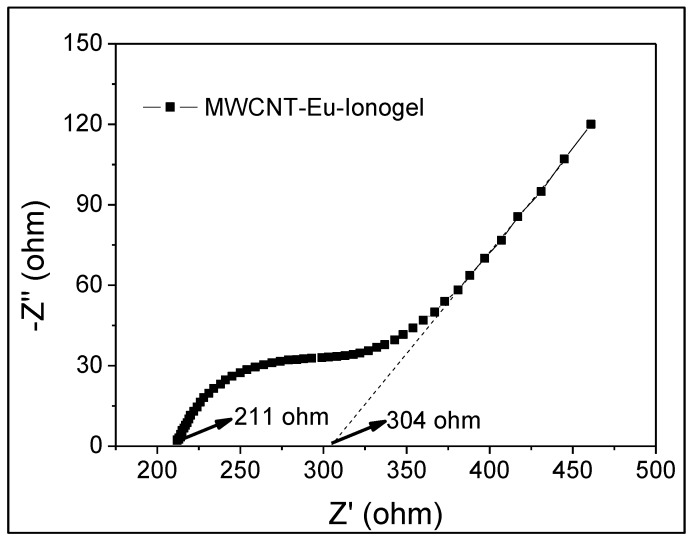
The Nyquist plot of the MWCNT-Eu-Ionogel equipped electrode.

To further understand the resistance of this composite ionogel, EIS analysis was performed in the frequency range of 0.01–100 kHz with an amplitude of 5 mV (Figure 6). For this measurement, the sample was sandwiched between two copper electrodes with an area of 1.5 cm^2^, and the thickness of the ionogel was calculated to be about 2 mm. The measurement temperature was kept at 25 °C. The Nyquist plots of the electrode were composed of straight line at low-frequency and depressed semicircle in the high-frequency region, which can be identified as the representative of ion diffusion and charge transfer characteristics. The interfacial charge transfer resistance in the high frequency region was 211 Ω. The slope of the curve corresponded to the Warburg impedance, which represented diffusive impendence of the ionic liquid onto the electrode surface. The analysis revealed that the embedded ionic liquids ensured that the ionogels have a certain level of conductivity.

## 4. Conclusions

In conclusion, two novel luminescent ionogels materials were designed and synthesized by introducing the lanthanide (Eu^3+^/Tb^3+^) complexes functionalized MWCNTs into a PMMA confined ionic liquid hybrid host system through a simple and easy, in situ thermal-curing reaction method. Therefore, the obtained luminescent materials are composites of ionic liquids, PMAA, lanthanide complexes, and MWCNTs. The lanthanide complexes modified MWCNTs were homogeneously dispersed in the ionogel matrices, to produce an intense red and green light emission from the europium and terbium complexes, respectively. The results show that the ionogels exhibit viscous, flexible, conductive, and outstanding photoluminescent properties, all of which qualify them as having great potential as applied materials in optical/electrical devices such as OLEDs and solar concentrators. This work has proven that the MWCNTs are excellent nanocarriers for luminescent rare earth complexes. The synthetic process may be applied to prepare other rare earth complexes-modified nanocarrier doped luminescent hybrid materials, that are expected to be promising optical/electrical multifunction materials.

## Supplementary Materials

The following are available online at http://www.mdpi.com/2073-4360/10/10/1099/s1, Figure S1: SEM images of the MWCNT-Eu-Ionogel (A and B) and the MWCNT-Tb-Ionogel (C and D).
