# Supplementary material for: Multifunctional Ionogels Incorporated with Lanthanide (Eu3+, Tb3+) Complexes Covalently Modified Multi-Walled Carbon Nanotubes
Source: Polymers (Basel). 2018 Oct 5;10(10):1099. doi: 10.3390/polym10101099 (PMC6404038; doi:10.3390/polym10101099)
Supplement: Supplementary file 1 [file polymers-10-01099-s001.pdf]

Supporting information

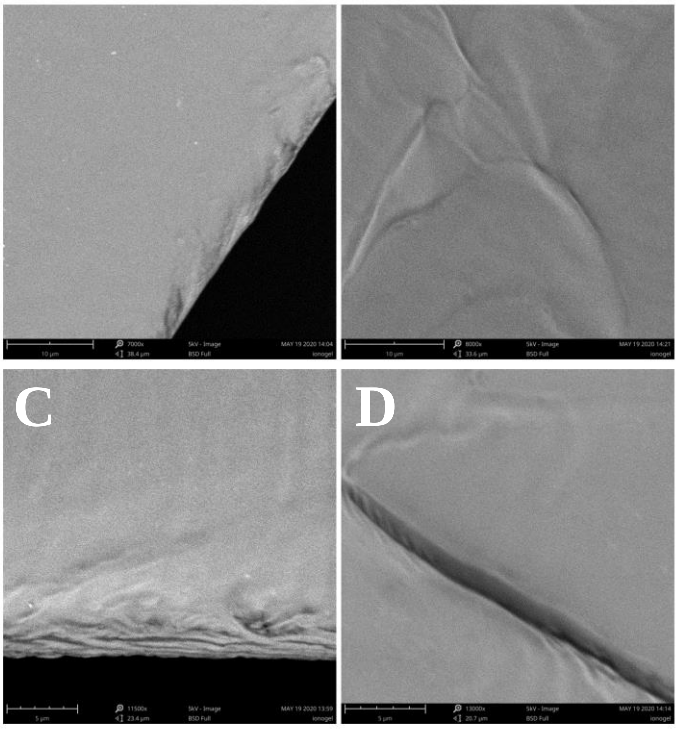

Fig. S1. SEM images of the MWCNT-Eu-Ionogel (A & B) and the MWCNT-Tb-Ionogel (C & D).

Calculation methods for the fluorescent quantum efficiency of MWCNT-Eu-Ionogel

The $^5D_0$ quantum efficiencies of all the europium complexes hybrid materials were estimated according to Juud-Ofelt theory. The detailed methods are listed below：

$$A_{0J} = A_{01} \times \frac{I_{0J}}{I_{01}} \times \frac{\nu_{01}}{\nu_{0J}}$$

$$A_r = \sum A_{0J} = A_{00} + A_{01} + A_{02} + A_{03} + A_{04}$$

$$\frac{1}{\tau} = A_r + A_{nr}$$

$$\eta = \frac{A_r}{A_r + A_{nr}}$$

A_{01} is the Einstein's coefficient of spontaneous emission between $^5D_0$ and $^7F_1$, the value of it is determined to be 50 s^{-1} theoretically; I_{0J} is the $^5D_0 \rightarrow {^7F_J}$ (J=0~4) transition intensities calculated from the peaks; and the ν_{0J} (J=0~4) is the energy barycenter of the corresponding emission peaks; A_r is the rate of radiative transition, and the A_{nr} is the one of non-radiative. Finally, the luminescence quantum efficiencies of europium complexes hybrid materials can be seen as equal to the ratio of A_r to value of (A_r + A_{nr}).
